# Functional and Morphological Characterization of Small and Large Steroidogenic Luteal Cells From Domestic Cats Before and During Culture

**DOI:** 10.3389/fendo.2019.00724

**Published:** 2019-11-14

**Authors:** Michał M. Hryciuk, Beate C. Braun, Liam D. Bailey, Katarina Jewgenow

**Affiliations:** ^1^Department of Reproduction Biology, Leibniz Institute for Zoo and Wildlife Research, Berlin, Germany; ^2^Department of Evolutionary Genetics, Leibniz Institute for Zoo and Wildlife Research, Berlin, Germany

**Keywords:** cat, luteal cells, steroid production, prostaglandins, gonadotropin reception

## Abstract

The current study aimed to isolate, culture and characterize small (SLC) and large (LLC) steroidogenic cells from the *corpora lutea* (CL) of non-pregnant domestic cats. Isolation of feline SLC was based on an enzymatic digestion of luteal tissue, whereas LLC were obtained by mechanical disruption of CL. To assess function of both cell types, progesterone secretion and mRNA expression of selected genes involved in steroid and prostaglandin synthesis were measured, as well as relative transcript abundance of hormone receptors and anti-oxidative enzymes, before and during culture. The cells were cultured for 3 or 5 days without gonadotropins. Isolated feline SLC and LLC had different sizes (12 ± 3 μm vs. 34 ± 5 μm, respectively), morphologies (amount of lipid droplets) and behaved differently in culture. SLC attached and proliferated or spread quickly, but lost their steroidogenic function during culture (significant decrease in progesterone secretion and expression of steroidogenic genes). The expression of receptors for gonadotropins and prolactin also decreased. Prostaglandin synthase (*PTGS2*) decreased steadily over time, whereas mRNA expression of *PGE2* synthase (*PGES*) increased. The gene expression of anti-oxidative enzyme glutathione peroxidase 4 (*GPX4*), also increased during culture, but not of superoxide dismutase 1 (*SOD1*). In comparison to SLC, LLC did not attach to culture plates, secreted more progesterone per inoculated cells and maintained steroidogenic function during culture. Expression of prostaglandin synthases (*PTGS2* and *PGES*) was almost non-detectable. The gene expression of hormone receptors for prostaglandin F2 alpha (*PTGFR*), gonadotropins (*LHCHR* and *FSHR*), and prolactin (*PRLR*), as well as of anti-oxidative enzymes (*GPX4, SOD1*), increased over time. To conclude, we successfully isolated and cultured different types of feline steroidogenic luteal cells and comprehensively characterized both isolated cell types. This knowledge can be used to better understand the CL lifecycle in felines more broadly, and the established cell cultures will provide a foundation for future studies on luteolytic and luteotrophic factors in the domestic cat, and for comparison with other feline species, particularly lynx.

## Introduction

The *corpus luteum* is a transient endocrine gland which forms on the mammalian ovary at the place of ovulation. The main function of *corpora lutea* (hereafter CL) is the production of progesterone, which is essential for the establishment and maintenance of pregnancy. CL are also known to synthesize and express receptors for hormones, e.g., sex steroids (1), prostaglandins (2), and gonadotropins (3).

Luteal cell cultures provide a valuable tool to study the functionality of CL, as previously described in many mammalian species like humans (4), rhesus monkeys (5), cows (6), pigs (7), sheep (8, 9), goats (10), rats (11), mice (12), dogs (13), and domestic cats (14). CL are composed of both small and large steroidogenic luteal cells, as well as non-steroidegenic cells such as fibroblasts, endothelial cells, pericytes, and immune cells (15). Small luteal cells (SLC) originate from *theca interna*, whereas large luteal cells (LLC) mainly originate from granulosa cells although they can also arise from SLC (16). Others have reported that theca and granulosa cell, *in vitro*, are able to transform into luteal cells (17, 18). The most prominent difference between SLC and LLC is their size, but the two types of luteal cells differ also in their steroidogenic capacity (4, 8, 11, 19–21), morphology (22), function (4), behavior in culture (11), and responsiveness to hormones (11, 20).

The domestic cat is a seasonal polyestrous species with ovulation induced by mating or by other intensive stimuli. Spontaneous ovulation, however, might also occur when there are no mating partners (23). Thus, CL that form following ovulation are present in either pregnant or non-pregnant luteal phases. The life cycle of pregnancy and the cycle CL was previously described by Dawson (24) and Amelkina et al. (25), and was divided into formation, development/maintenance and regression stages. At the formation stage, growth of the gland is caused by division of steroidogenic and non-steroidegenic cells, vascularization and hypertrophy of steroidogenic cells (24). As CL age, feline steroidogenic cells change their size, shape and degree of vacuolisation. Based on morphology and hormonal activity, there are no differences between similar stages of CL in pregnant and non-pregnant cycles (25). However, based on serum progesterone, the duration of pregnancy is 66 days (26), in contrast to 40 days for a non-pregnant cycle (27). The CL of pregnancy achieve their maximal size 10–16 days after coitus (24). The highest hormonal activity has been determined at around 21 days following ovulation and then decreases until parturition (28). In the case of a non-pregnant cycle, P4 serum concentrations also peak around 21 days post ovulation and drop to baseline at around day 40 (28).

In most mammalian species, CL usually regress to *corpora albicantia* after pregnancy or at the end of the luteal phase of the ovarian cycle. An exception is the so called “persistent CL” which can be found on the ovary outside of these periods. Persistent CL are considered a pathological disorder and are connected to hormonal disruption and infertility, e.g., in cows (29, 30). In contrast, physiologically persistent and hormonally active CL have been described in lynx (31, 32). The lynx CL persist on the ovary for at least 2 years (33) and continuously produce progesterone (P4) (31, 34) at a level comparable to the serum levels of domestic cats during early pregnancy (5–10 ng/mL) (28). It has been suggested that the permanent progesterone levels in lynxes prevent further ovulations and in doing so, turn a polyestrous cycle into a monoestrous pattern (33). This feature is unique within the feline family and demands comparative investigation of luteal function between lynxes and cats.

The aim of the current study was to establish a cell culture system for steroidogenic luteal cells from the domestic cat. We separated small (SLC) and large (LLC) luteal cells from domestic cat CL of development/maintenance stages and cultured them for up to 3 or 5 days. Both cell types were analyzed for basal progesterone secretion (without gonadotropin stimulation) *in vitro* and RNA expression of selected genes involved in steroidogenesis and prostaglandin synthesis as well as hormone receptors and anti-oxidative enzymes before and during culture. The characterized cell culture system will provide a foundation for future studies on potential luteolytic and luteotrophic factors in the domestic cat, and for comparison to lynx species, especially with regards to the function of persistent CL.

## Materials and Methods

This study was approved by the Internal Committee for Ethics and Animal Welfare of the IZW (2017-02-02). All chemicals used in these experiments were purchased from Merck KGaA, Darmstadt, Germany unless otherwise stated.

### Ovaries and *Corpora lutea*

Ovaries were obtained from non-pregnant domestic cats after routine ovariectomy at animal shelter of Berlin. The ovariectomia were not related to the purpose of the experiment. Ovaries were transported to the laboratory in HEPES-MEM medium, supplemented with 3 g/L BSA and 1x Antibiotic Antimycotic Solution in 50 mL tubes (Sarstedt AG & Co. KG, Nümbrecht Germany). Upon arrival, ovaries were isolated from surrounding tissues and washed twice in Dulbecco PBS (DPBS), and checked for the presence of CL indicating a non-pregnant luteal cycle. CL were isolated and washed in fresh DPBS. Half of one CL per animal was fixed in Bouin solution and used for identifying the stage of the luteal life cycle as described by Amelkina (25). For the purpose of the experiments, luteal cell cultures from 17 queens were made, of which 9 were defined as development/maintenance stage. Due to the small size of cat CL, 0.3 × 10^6^ SLC and 0.1 × 10^6^ on average LLC were able to be isolated from one CL. Thus, a full experimental trial including gene expression analysis on both cell types obtained from the same domestic cat was only possible in six cases. The data from these selected experiments (*n* = 3 for experiment A; *n* = 3 for experiment B) were compiled for statistical analysis. All other experiments contributed to the microscopic and steroidogenic characterization (see below) of SCL and LLC.

### Experimental Design

For each experiment (A and B), three independent cell culture trials (each trial from one cat) were performed. From a pair of ovaries, CL were equally pooled into two groups to isolate small and large luteal cells resulting in two independent cell suspension of SLC and LLC.

**Figure 1:**
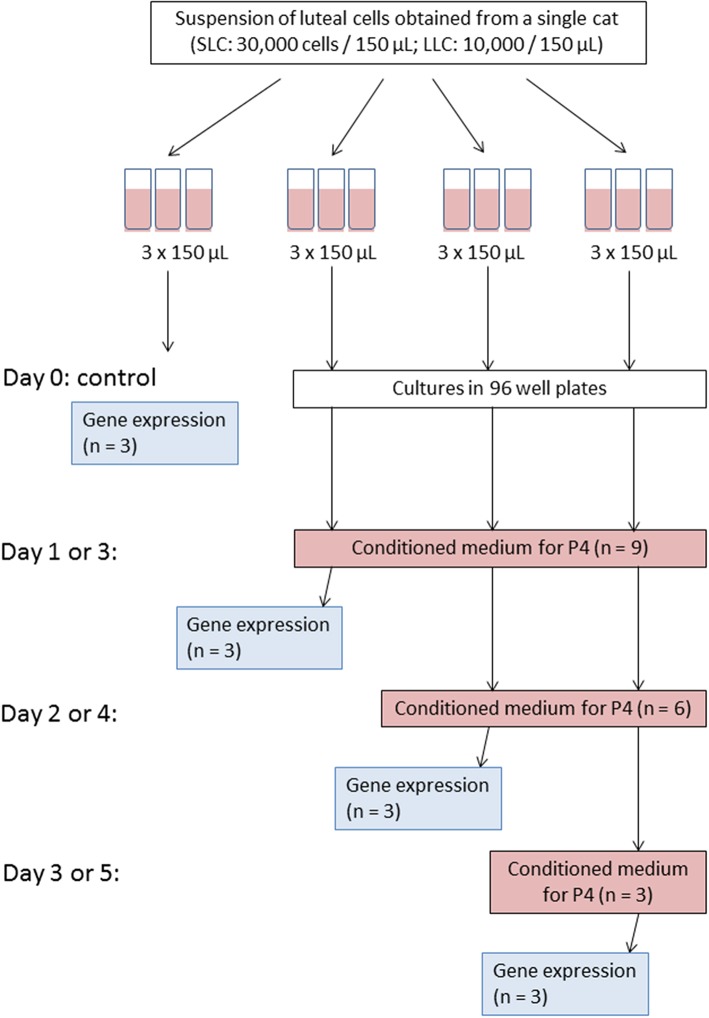
Scheme of experiment for one culture trial. Please note that for Experiment A medium change and cell harvest were performed on day 1, 2, and 3; whereas for Experiment B on day 3, 4, and 5, accordingly.

Initially, each cell suspension was set on a certain cell concentration (see below) and divided into 12 technical replicates of 150 μL (Figure 1); three of them were immediately used as a control. The control samples were subjected to gene expression analysis (see below). In the Experiment A, the remaining nine replicates were aliquoted into 96-well plate and were cultured for 1, 2, or 3 days, respectively. On each day of culture, conditioned medium from all replicates was collected for progesterone analysis (see below), and cells were harvested from three replicates for gene expression analysis (see below). Fresh medium was added to the remaining wells of the 96-well plate. In Experiment B, the cell culture was performed for 3, 4, and 5 days. Accordingly, medium changes for progesterone analysis and cell harvest was performed on day 3, 4, and 5, respectively.

Because of this setup, progesterone secretion was measured in 9, 6, and 3 wells on the first, second and third time point, respectively. For relative transcript abundance analysis, three measurements were taken from freshly isolated cells and at each of the three time points of the culture period.

### Isolation of Small Luteal Cells (SLC)

Small luteal cells were isolated by a modified enzymatic method (14). CL were placed into a Petri Dish (60 × 10 mm, Thermo Fisher Scientific, Dreieich, Germany) containing medium I (HAM's F12 and MEM Eagle Medium 1:1 supplemented with 0.055 mg/mL gentamicin and 5% FBS), chopped into small pieces and transferred on a 40 μm cell strainer (VWR International, Dresden, Germany), which was placed into another Petri dish. Pieces were covered with medium I supplemented with 0.1% collagenase (types I and II; SERVA Electrophoresis GmbH, Heidelberg, Germany) and 0.005% DNAse I and digested on the strainer for 55 min at 39°C. Thereafter, pieces were gently smashed through the strainer and the obtained suspension was agitated by pipetting and transferred to a glass tube for centrifugation (7 min at 1,000 × g). The cell pellet was resuspended in 2 mL fresh medium I and placed on 40% percoll solution (with DPBS) in a glass tube. After centrifugation for 7 min at 1,000 × g, cells were collected from the interphase between medium I and percoll solution, transferred to 1.5 mL reaction tubes and centrifuged at 500 × g for 4 min. The obtained cell pellet was resuspended in fresh medium I, the cell concentration was determined and set to 200,000 cells per mL (30,000 per 150 μL).

### Isolation of Large Luteal Cells (LLC)

Large luteal cells were isolated by a mechanical method. CL were chopped and smashed through a cell dissociation sieve (380 μm) placed into a Petri Dish (60 × 15 mm, Thermo Fisher Scientific) with medium II (HAM's F12 and MEM Eagle Medium, 1:1 (v:v) supplemented with 0.055 mg/mL gentamicin and 1% FBS). To remove pieces of tissue, cell suspension was filtered through cell strainer (35 μm) connected to a 5 mL polystyrene round bottom tube (BD Biosciences Discovery Labware, Canaan, USA) and centrifuged for 7 min at 1,000 × g. The cell pellet was resuspended with medium II and placed on 20% percoll solution (with DPBS) in a glass tube and centrifuged for 7 min at 1,000 × g. Large luteal cells were collected from the interphase between medium II and percoll solution, transferred to 1.5 mL reaction tubes and washed by centrifugation for 4 min at 500 × g. After re-suspension in fresh medium II, the cell concentration was determined and set to 67,000 cells per mL (10,000 per 150 μL).

### Cell Culture

Tissue Culture Plates (96-well, Sarstedt) were coated with 15 μl of 0.2% Collagen R (SERVA) diluted 1:10 with DPBS. The collagen solution was evenly distributed on the bottom of wells and left to dry under the hood for 7 h. Coated plates were stored at 6°C until use.

Small and large luteal cells were cultured separately in 150 μL medium I and II, respectively, (see cell concentration above) at 39°C, 5% CO_2_. Concentration of FBS in medium for SLC and LLC was different because this ingredient may inhibit responsiveness of the steroidogenic cells toward LH (35). Our aim was to establish conditions for future functional studies; therefore, we have tested medium with 1 and 5% of FBS on SLC and LLC during culture period. In the preliminary experiments, SLC required minimum 5% of FBS in medium. Lower concentration of FBS caused that SLC did not proliferate or spread as fast as in wells with 5% of FBS. There was also much more cell debris, which may indicate on cells death caused by unsuitable medium. For LLC, no difference was observed between the cells in a medium supplemented with 1 and 5% of FBS.

Medium change was performed by replacing 130 μL conditioned medium by freshly prepared medium. For SLC, collected medium was centrifuged for 4 min at 500 × g and then transferred to a new reaction tubes. LLC are not adherent cells, therefore collected medium was centrifuged, then supernatant was transferred to a new reaction tube and a potential pellet with LLC was resuspended with fresh medium and returned to corresponding well. On the day of medium change, the conditioned medium was frozen for hormone analysis at −20°C. In addition, cells from three culture wells were harvested for mRNA analysis. To collect cells from the dish, they were overlayed with Trypsin-EDTA solution (100 μL, 15 min at 39°), and transferred to 1.5 mL reaction tubes contained 100 μL medium. Then, wells were washed twice with medium 500 × g for 4 min. Harvested cells were stored in RNAlater at −20°C, until RNA isolation.

### Microscopic Analysis and Cell Measurement

Luteal cell cultures were analyzed under an Axiovert 200M microscope (Carl Zeiss, Oberkochen, Germany) equipped with a ProgRes^®^ camera using the CapturePro 2.10.01 program (JENOPTIK Optical systems GmbH, Berlin, Germany). Digital photos were obtained from freshly isolated SLC and LLC, and were used to determine mean cell diameter with the help of imaging software (cell∧D, Olympus Soft Imaging Solutions GmbH, Münster, Germany). For each cell suspension (*n* = 7) a minimum of 10 cells were measured to characterize size of isolated cells.

### HSD3B Assay for Identification of Steroidogenic Luteal Cells

For identification of steroidogenic luteal cells, a modified 3β-hydroxysteroid dehydrogenase (HSD3B) activity assay was performed (36) on cell cultures where there were not enough isolated cells for a complete experimental trial. In brief, luteal cells were fixed with 1% formaldehyde in DPBS (15 min, 39°C). Thereafter, they were washed twice in DPBS and incubated for 24 h in staining solution (PBS containing 0.25 mM nitrotetrazolium blue chloride (NBT), 0.1% BSA, 1.5 mM β-nicotinamide adenine dinucleotide hydrate (NAD+), 0.2 mM pregnenolone and 2 mM EDTA). After incubation, cells were washed in DPBS and analyzed under the microscope (Carl Zeiss). Staining control was performed by adding trilostane (2 mM) to the staining solution. Trilostane inhibits specifically the activity of 3β-hydroxysteroid dehydrogenase (37).

### Progesterone Determination by Enzyme-Linked Immunosorbent Assay (ELISA)

Progesterone extraction was performed with modifications as described before (38). In detail, medium samples (100 μL) obtained after each medium change were transferred to test tubes (16 × 130 mm, Carl Roth GmbH + Co. KG, Karlsruhe, Germany) followed by addition of 900 μL of PBS and 2.5 mL of methyl-tert-butyl ether/petroleum ether (v:v; 3:7). After shaking for 30 min, the tubes were placed into a freezer (−80°C) for 15 min. Subsequently, the organic phase was decanted into a new tube (16 × 100 mm, Corning Incorporated, New York, USA) and evaporated under a stream of N_2_ for 10 min at 50°C. Thereafter, samples were quickly dissolved in 80 μl of 100% methanol, and diluted with 120 μl of distilled water. Recovery values for extraction control samples were in a range 92.3–111.8% for medium I and 90.3–107.9% for medium II. The samples were stored at −20°C until determination of progesterone by ELISA.

Progesterone (P4) analyses were carried out with an in-house microtiter plate enzyme immunoassay as described earlier (39) using a commercial P4 antibody (Sigma P1922, raised in rats to progesterone) and 4-pregnen-3,20-dione-3-CMO-peroxidase label. The cross-reactivities to other steroids were as follows: 4-pregnen-3,20-dione (progesterone), 100%; 5a-pregnan-3,20-dione, 31%; 5a-pregnan-3b-ol-20-one, 18%; 5-pregnen-3b-ol-20-one, 12%; 4-pregnen-3aol-20-one, 4.2%; <0.1% for 5b-pregnan-3a,20adiol, 4-pregnen-20a-ol-3-one, 5b-pregnan-3a-ol-20-one, 5a-pregnan-20a-ol-3-one, 5a-pregnan-3a,20 a-diol, 5a-pregnan-3b,20a-diol, testosterone, estradiol, and cortisol. For ELISA measurements, we used 20 μL of sample extract, which was dispensed on a plate with 100 μL of an enzyme dilution and then 100 μL of antibody solution was added. Intra assay coefficients for two biological samples with low and high concentration were 12.0 and 4.5%, respectively. The respective inter assays were 13.8 and 10.0%. We confirm linearity of extraction method and parallelism of diluted samples.

### Sequence Analysis of Genes of Interest

At the beginning of the present study, sequence information was confirmed for some genes of interest, while only predicted sequences were known for others. Thus, in order to design primers suitable for real-time PCR, the predicted sequences must be confirmed. Feline gene sequences were previously confirmed for prostaglandin endoperoxide synthase 2 (*PTGS2*) (40), prostaglandin E2 synthase (*PTGES*) (41), cytochrome P450 family 11 subfamily A polypeptide 1 (*CYP11A1*) (38), 3β-hydroxysteroid dehydrogenase type 1 (*HSD3B1*) (38), prostaglandin E receptor 2 (*PTGER2*) (2), and prostaglandin F receptor (PTGFR) (2). However, gene sequences need to be confirmed for luteinizing hormone receptor (*LHCGR*), prolactin receptor (*PRLR*), follicle stimulating hormone receptor (*FSHR*), glutathione peroxidase 4 (*GPX4*), and superoxide dismutase 1 (SOD1) (see GenBank accession numbers for all genes in Table 1). To accomplish this, total RNA was isolated from feline *corpora lutea* tissues according to the innuSPEED Tissue RNA/innuPREP DNase I Digest Kit (*PRLR, FSHR, GPX4, SOD1*; Analytik Jena AG, Jena, Germany) or the Precellys Tissue RNA/peqGOLD DNase I Digest Kit (*LHCGR*; Peqlab, part of VWR International GmbH) as described in Amelkina et al. (43). Reverse transcription of total RNA into single-stranded cDNA (ss cDNA) was performed with the RevertAid First Strand cDNASynthesis Kit (Thermo Scientific, Schwerte, Germany). For the polymerase chain reaction (PCR) primers were purchased from BioTeZ Berlin Buch GmbH (Berlin, Germany) or Merck KGaA. Primer information is listed in Table 1. Based on feline ss cDNA templates partial cDNA sequences were amplified using the Expand High FidelityPLUS PCR system (Roche Diagnostics Deutschland GmbH, Mannheim, Germany), as described before (44). The PCR conditions were: 94°C for 2 min; followed by 35 cycles of denaturation (94°C) for 30 s (*LHCGR, PRLR*–sequence part 1) or 60 s (*PRLR*–sequence part 2, *FSHR, GPX4, SOD1*), annealing (see temperatures in Table 1) for 30 s (*LHCGR, PRLR-1*) or 60 s (*PRLR-2, FSHR, GPX4, SOD1*), elongation (72°C) for 30 s (*LHCGR*), 60 s (*PRLR*–sequence part 1), 120 s (*PRLR*–sequence part 2, *GPX4, SOD1*), or 150 s (*FSHR*); and a final elongation at 72°C for 7 min. For *PRLR-1* and *FSHR* the purified PCR products were ligated to the pJET 1.2 vector (Thermo Scientific) and transformed in DH5alpha cells (Life Technologies GmbH, Darmstadt, Germany). The *LHCGR* product was ligated into the pCR4-TOPO TA vector and transformed in TOP10 cells (both Life Technologies GmbH). The PCR-products of *PRLR*–sequence part 2, *GPX4* and *SOD1* and positive clones of *FSHR, LHCGR* and *PRLR*–sequence part 1 were sequenced by the Services in Molecular Biology GmbH (Dr. M. Meixner, Brandenburg, Germany).

**Table 1:** Sequence of primers used in real-time PCR and for sequence analysis.

**Gene**	**GenBank accession**	**Species**	**Primer sequence 5^**′**^−3^**′**^**	**TA [^**°**^C]**	**Product size [bp]**	**Use**	**References**
*betaActin*	AB051104	*Felis catus*	qfw: GAG CAG GAG ATG GCC ACG qrv: CTC GTG GAT GCC ACA GGA	62	159	a	(42)
*GLS*	JQ424891	*Felis catus*	qfw: TCC AGC TAT GCT CCA TTG AAG T qrv: TGC AGG AAG ACC AAC ATG G	61	196	a	(38)
*TBP*	JQ424890	*Felis catus*	qfw: AGA GAG CCC CGA ACC ACT G qrv: TTC ACA TCA CAG CTC CCC AC	62.5	182	a	(38)
*CYP11A1*	JN165033	*Felis catus*	qfw: CTT CCG GAA CCT GGG CTT qrv: GCA GCG TCC ACC CTC TCT A	61.5	240	a	(38)
*HSD3B1*	JN127378	*Felis catus*	qfw: GCC CTA CAG GAC CCC AAG A qrv: TTC CAG CAG GAA GGC AAG C	62.5	182	a	(38)
*PTGS2/ COX2*	EF036473	*Felis catus*	qfw: CAG GAG GTC TTT GGT CTG G qrv: CCT GCT CGT CTG GAA CAA	58	135	a	(2)
*PGES/ PTGES*	GU059259	*Felis catus*	qfw: TCG CTG CCT CAG AGC CCA qrv: TAG GCC ACG GTG TGC ACC	66	153	a	(2)
*PTGER2*	EF177829	*Felis catus*	qfw: GAG GGG AAA GGC TGT CCA qrv: GCA AAA ATT GTG AAA GGC AAG	56.5	103	a	(2)
*PTGFR*	AF272340	*Felis catus*	qfw: GCT GGA GTC CAT TTC TGG TG qrv: CCA CGT TGC CAT TCG AAG	61	104	a	(2)
*LHCGR*	KP826762^*^	*Felis catus*	qfw: CCT GGT GTA CAT CGA GCC T qrv: GGA TTC GTT ATT CAT CCC TTG fw: CAC TCA CCT ATC TCC CTA TC rv: GGA GTG TCT TGG GTG AGC	57.5 53	199 861	a b	This study
*PRLR*	MH882487^*^/ MH882488^*^	*Felis catus*	qfw: GCT CAC ACT CCA GTA CGA AA qrv: TCT GTC CTG GAT ATA AGC TGA fw1: CTG ATA CAT TTC CTG TAG AAA GAG rv1: TCT GTC CTG GAT ATA AGC TGA fw2: GCT CAC ACT CCA GTA CGA Aa rv2: CCA ATC GTT CCA TTA ATC AAG C	60.5 51 55	112 635 1414	a b b	This study
*FSHR*	MH882490^*^	*Felis catus*	qfw: GCA AAT GTG TTC TTC AAC CTG T qrv: GGA GGT TGG GAA GGT TCT G fw: CTC AGG ATG TCA TCA TCG G rv: GTG AGA CTT CAG TTA TCC TTT G	59.5 53	106 2045	a b	This study
*GPX4*	MH882486^*^	*Felis catus*	qfw: CTT GCA ACC AGT TCG GGA G qrv: CTT GGG CTG GAC TTT CAT CC fw: CTG TGC TCA GTC CAT GCA C rv: CTT GTG GAG CTA GAG GTA G	58.5 53	154 497	a b	This study
*SOD1*	MH882489^*^	*Felis catus*	qfw: GAG AGG CAT GTT GGA GAC CT qrv: GTC ATC TCG TTT CTC GTG GAC fw: GAG CAT GGA TTC CAC GTC C rv: CTC AGA TCG CAT CCT AGG G	59.5 53	144 364	a b	This study

* *Analyzed in this study*.

*a, expression study; b, sequence analysis*.

### mRNA Expression Analysis by Real-Time PCR

Isolation of total RNA was performed by NucleoSpin^®^ RNA Plus XS (Macherey-Nagel GmbH & Co. KG, Berlin, Germany) according to manufacturer's manual. Concentration and purity of isolated RNA was measured on NanoDrop™ 2000c (Thermo Fisher Scientific). 13 μl of isolated RNA solution was reverse transcribed into cDNA with PrimeScript RT Reagent Kit (TAKARA BIO INC., Kusatsu, Japan) according to the manufacturer's manual, with the exception that Oligo dT primers (25 pmol) and random hexamers (50 pmol) were both used per reaction.

Real-time PCR was performed in CFX96 Real-Time PCR Detection System (Bio-Rad Laboratories GmbH, Munich, Germany) on 96-Well PCR Plates (Bio-Rad) as described before (38). Diluted cDNA was analyzed in a 10 μl reaction volume containing SsoFast EvaGreen Supermix (Bio-Rad) and primers (f.c. 500 nM each). Reactions conditions were: 98°C for 2 min and 40 cycles at 98°C for 8 s and 8 s for primers annealing at different temperatures. Detailed information about primers and annealing temperatures is presented in a Table 1. Bio-Rad CFX Manager 3.1 Software was used for quantification data. Serial dilutions of PCR products (*LHCGR, FSHR, GPX4*) or recombinant plasmid carrying desired genes (all other genes) were used for calibration. A normalization factor for qPCR analysis of SLC samples was calculated based on mean values of relative transcript abundance for beta Actin (*BACT*), glutaminase (*GLS*), and TATA-Box Binding Protein (*TBP)* using qbasePLUS software (Biogazelle, Zwijnaarde, Belgium) (45). For LLC only *BACT* values were used for normalization as GLS and TBP were not detectable in most samples. Mentioned reference genes were previously described by Zschockelt et al. (46) as suitable reference genes for luteal tissue. No template control (NTC) and no reverse transcriptase control (NRT) samples were included in analysis.

### Statistical Analysis

All data presented for P4 analysis and gene expression analysis were depicted as mean ± standard deviation for all replicates within same day of experiment A and B and for SLC and LLC.

Each cell culture was isolated from an individual cat and may therefore have differed in their baseline P4 concentration and transcript abundance, meaning that different measurements taken from the same cell culture could not be fully independent. To account for the lack of independence between technical replicates, P4 concentration and transcript abundance were mean centered within each cell culture. The mean value of P4 concentration and transcript abundance was subtracted from each individual measurement, so that all measurements were adjusted to be relative to the mean value of that particular cell culture. With this approach, all statistical tests considered relative changes within a cell culture between time periods rather than absolute change in P4 concentration and transcript abundance. This approach accounts for any differences in absolute P4 concentration and transcript abundance that may be caused by differences in the individual cat from which cells were isolated.

All analyses were performed in R (R: A language and environment for statistical computing (2018); R Foundation for Statistical Computing, Vienna, Austria; v. 3.5.0). Relative P4 concentration and transcript abundance between time periods was analyzed using a Kruskal-Wallis rank sum test followed by a *post-hoc* pairwise Wilcoxon rank sum test for comparison between group levels using the Benjamini-Hochberg adjustment for multiple testing (47). Analyzing day as a categorical rather than continuous variable gave the possibility to detect non-linear changes in P4 concentration and transcript abundance over time. *P*-values lower than 0.05 were considered statistically significant. Chi-square test statistics and degrees of freedom are provided in result summary tables (Table 2, Tables S1, S2).

**Table 2:** Progesterone concentration in cell culture medium determined by EIA.

	**Type of cells**	**Medium obtained from culture period:**					
		**Day 0–1**	**Day 1–2**	**Day 2–3**	***P*-value**	**Chi-squared test statistic**	**df**
Experiment A	SLC	4706 ± 2902^a^ pg P4/mL	122 ± 180^b^ pg P4/mL	345 ± 153^b^ pg P4/mL	<0.0001	40.15	2
		5.7 ± 3.46^a^ pg P4/ng RNA	0.7 ± 0.7^b^ pg P4/ng RNA	0.16 ± 0.09^b^ pg P4/ng RNA	<0.001	17.85	2
	LLC	5247 ± 2470^a^ pg/mL	550 ± 235^b^ pg/mL	206 ± 82^b^ pg/mL	<0.0001	40.29	2
		6.55 ± 3.61^a^ pg P4/ng RNA	0.84 ± 0.36^b^ pg P4/ng RNA	0.28 ± 0.19^b^ pg P4/ng RNA	<0.001	17.91	2
		**Day 0–3**	**Day 3–4**	**Day 4–5**			
Experiment B^*^	SLC	2608 ± 999 pg/mL	48 ± 58 pg/mL	288 ± 185 pg/mL			
	LLC	14431 ± 9447 pg/mL	1867 ± 1851 pg/mL	509 ± 534 pg/mL			

*SLC, small luteal cells; LLC, large luteal cells. Mean values ± standard deviation correspond to 30,000 SLC and 10,000 LLC seeded into wells at the day of initiating cell culture. For each experiment and each cell type, three cell preparations were used to generate P4 concentrations. Values in a second row, describe amount of progesterone produced in one well-normalized with total RNA value for the corresponding well. Non-parametric Kruskal-Wallis test was used for statistical analysis. Chi squared—refers to test statistics and df indicates degrees of freedom. Different superscripted letters indicate significant differences between groups, based on pairwise comparison Wilcoxon rank sum test*.

* *For Experiment B, statistical analysis and normalization values ware not performed because the first values describe amount of progesterone accumulated over 3 days while other values describe amount of P4 accumulated over 1 day period*.

The relative transcript abundance for selected genes was depicted as vertical box plot by plotting medians and percentiles (Sigma.Plot 10.0 Systat software GmbH, Erkrath, Germany).

## Results

### Characterization of Luteal Cells in Culture

Within isolated SLC suspension, 99.3% of cells had diameter smaller or equal to 20 μm, while in isolated LLC, the percentage of cells with diameter >20 μm was 99.0%. Sometimes suspension with isolated cells contained cell debris which could not be removed by washing and were not counted.

**Figure 2:**
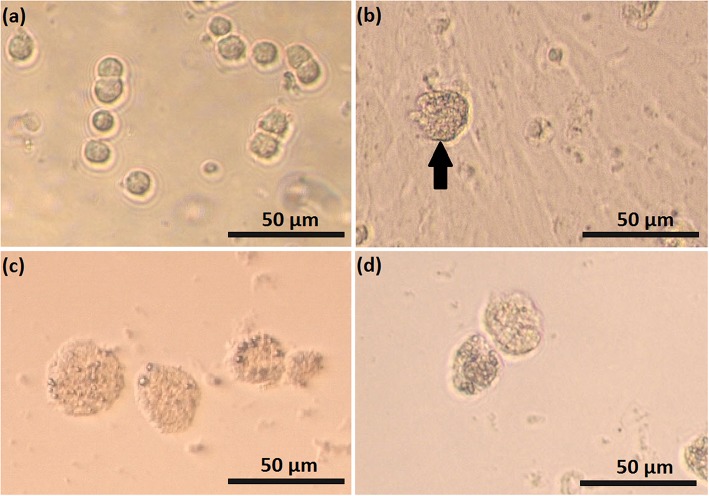
Photomicrographs of small (SLC) and large (LLC)–luteal cell. SLC **(a)** and LLC **(c)** after seeding to culture dish; **(b)** SLC culture at day 5, black arrow indicate on a cell which may have differentiated, **(d)** LLC culture at day 5.

**Figure 3:**
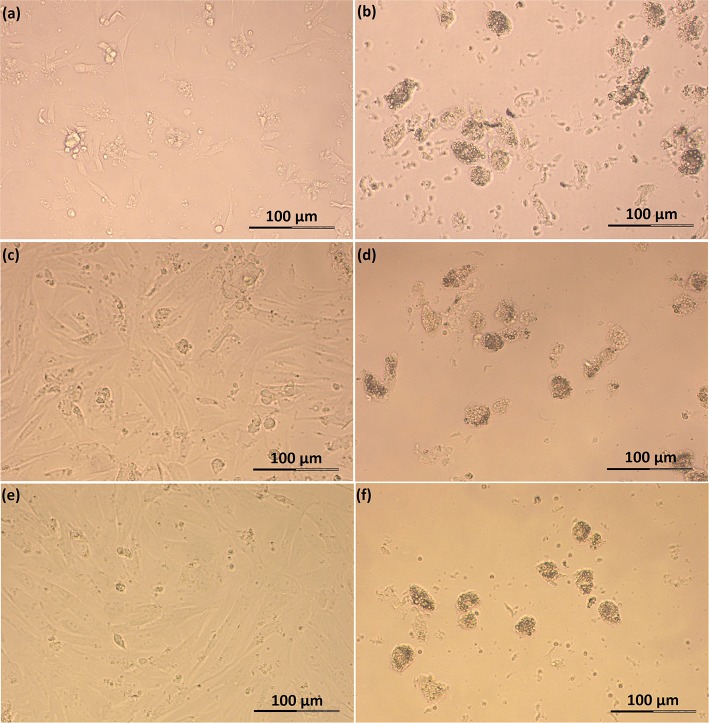
Photomicrographs of SLC and LLC in cell culture during experiment A: **(a)** SLC at day 1, **(b)** LLC at day 1, **(c)** SLC at day 2, **(d)** LLC at day 2, **(e)** SLC at day 3, and **(f)** LLC and day 3. The series of photos are from one individual.

**Figure 4:**
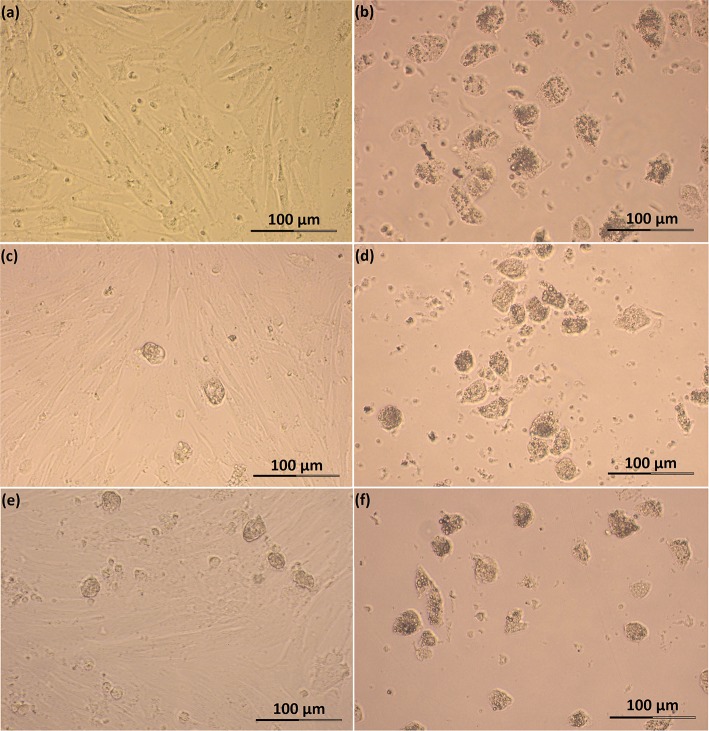
Photomicrographs of SLC and LLC in cell culture during experiment B: **(a)** SLC at day 3, **(b)** LLC at day 3, **(c)** SLC at day 4, **(d)** LLC at day 4, **(e)** SLC at day 5, and **(f)** LLC and day 5. The series of photos are from one individual.

Feline SLC and LLC varied in morphology and behaved differently in cell culture (Figure 2). Isolated SLC had an average diameter of 12 ± 3 μm and were round (Figure 2a). After 24 h of culture, the cells attached to the bottom of the culture well and became elongated (Figures 3a,c,e, 4a,c,e). Proliferating or spreading cells covered most of the bottom of a well at around the fourth day of culture. In experiment B, at around the fourth day of culture, visual observations showed that some of the small luteal cells accumulated lipid droplets and detached from the culture plate (Figures 2b, 4c,e). The detached cells looked similar to LLC, as they were large and round. These observations were made only occasionally.

Isolated LLC were almost three times larger than SLC with an average diameter of 34 ± 5 μm (Figure 2c). They were round to oval and contained a high amount of lipid droplets. Based on microscopic analysis during the cell culture period, the number and size of lipid droplets in LLC seemed not to change (Figures 3b,d,f, 4b,d,f). LLC did not attach to the cell culture plate, and did not proliferate (Figure 2d). According to photographs taken every day, the number of LLC seemed to decrease during the culture period, possibly due to cell death and cell loss by medium change (Figures 3b,d,f, 4b,d,f).

### Identification of Steroidogenic Luteal Cells

**Figure 5:**
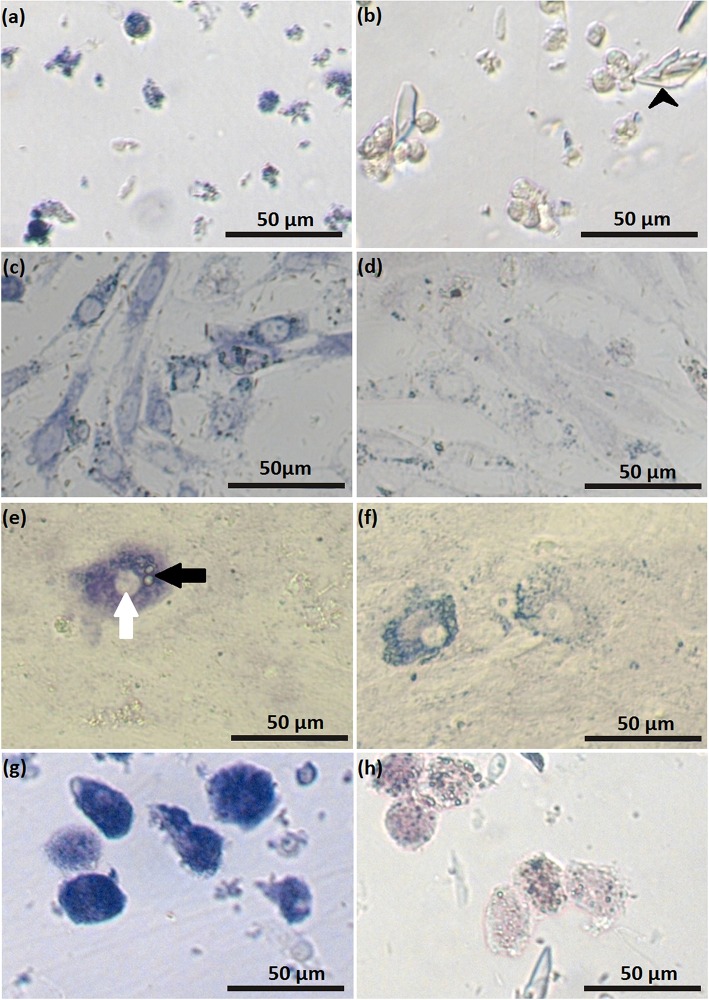
Luteal cells stained for activity of 3β-hydroxysteroid dehydrogenase: freshly isolated small (SLC) luteal cells **(a)**; control staining for freshly isolated SLC **(b)**, black arrowheads indicate crystals of trilostane, which could not be removed from wells with non-adherent cells; SLC at day 2 of cell culture **(c)**; control staining for SLC at day 2 of cell culture **(d)**; SLC at day 5 of cell culture **(e)**, white arrow points to the nucleus, black arrow points to lipid droplets; staining control for SLC at day 5 of cell culture **(f)**; freshly isolated large luteal cells (LLC) **(g)**; control staining freshly isolated LLC **(h)**. In control samples, activity of 3β-hydroxysteroid dehydrogenase was blocked by trilostane.

Suspensions of stained, freshly isolated SLC and LLC clearly indicate their steroidogenic capacity (Figures 5a,g, respectively). For SLC around 68% of isolated cells was intensively stained or had partial staining which allowed us to distinguish them from cells without any steroidogenic activity. In contrast to this, all isolated LLC were stained. Among them, around 85% of isolated LLC were characterized by very high steroidogenic activity what was expressed by dark blue color of cells. Another 15% of isolated cells have lower steroidogenic activity, what could be distinguished by lighter blue color. There was still visible difference between LLC with low steroidogenic activity and cells in control staining group.

Small luteal cells were also stained for activity of HSD3B during cell culture. On day 2, most of the attached cells expressed steroidogenic activity (Figure 5c, Figure S1), while on day 5, the cells were characterized by partial staining. Remarkably, some cells were intensively colored dark blue and contained round nuclei and numerous large cytoplasmic lipid droplets (Figure 5e).

Trilostane was used to specifically block the activity of 3β-hydroxysteroid dehydrogenase, resulting in reduced enzyme activity (Figures 5b,d,f,h). The remaining staining after trilostane treatment might indicate other NAD^+^-dependent metabolic reactions. In contrast to cell monolayers (SLC on day 5 of culture), crystals of trilostane could not be removed by washing from the cell suspensions.

### Progesterone Production

For experiment A, there was a significant difference in P4 concentration between time periods in both SLC and LLC cultures (Table 2; *p* < 0.001). In all cases, *post-hoc* pairwise comparison of groups showed that P4 concentration was significantly higher during the first period than either of the later time periods (Table 2). Our presented hormone concentrations do not consider changes in cells number during cell culture period. For experiment B, the first measures describe progesterone concentration accumulated over 3 days, while the following measurements describe progesterone accumulated over 1 day period, therefore statistical analysis for this experiment was not performed.

For SLC cultures, P4 concentration in experiment A did not statistically increase between the middle and final time period due to very large variations in cell preparations (Table 2). In comparison, P4 concentration in LLC was decreasing progressively between time periods (Table 2). However, neither of these differences was significant in *post-hoc* pairwise comparisons.

Concentration of secreted progesterone was higher in the medium obtained from LLC, than from SLC. Thus, LLC produce more progesterone per cell than SLC, because initial number of steroidogenic cells was three times higher for SLC than for LLC.

### Expression Analysis by Relative Transcript Abundance

**Figure 6:**
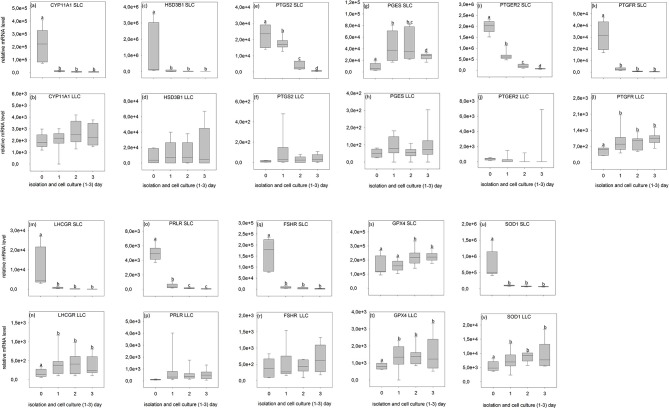
Relative mRNA level of analyzed genes in SLC (small luteal cells) and LLC (large luteal cells) obtained directly after isolation (Day 0) and after 1–3 days of culture (Experiment A). **(a,b)** *CYP11A1*, Cholesterol Side-Chain Cleavage Enzyme, **(c,d)** *HSD3B1*, 3β-hydroxysteroid dehydrogenase type 1, **(e,f)** *PTGS2*, prostaglandin-endoperoxide Synthase 2, **(g,h)** *PGES*- Prostaglandin E synthase, **(i,j)** *PTGER2*, prostaglandin E receptor 2, **(k,l)** *PTGFR*, prostaglandin F receptor, **(m,n)** *LHCGR*, luteinizing hormone/choriogonadotropin receptor, **(o,p)** *PRLR*, prolactin receptor, **(q,r)** *FSHR*, follicle stimulating hormone receptor, **(s,t)** *GPX4*, glutathione peroxidase 4, **(u,v)** *SOD1*, superoxide dismutase 1. Small letters indicate significant differences between groups, based on pairwise comparison Wilcoxon rank sum test.

**Figure 7:**
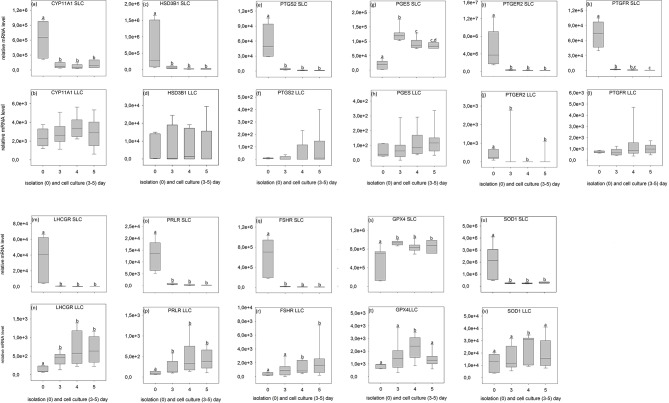
Relative mRNA level of analyzed genes in SLC (small luteal cells) and LLC (large luteal cells) obtained directly after isolation (Day 0) and after 3–5 days of culture (Experiment B). **(a,b)** *CYP11A1*, Cholesterol Side-Chain Cleavage Enzyme, **(c,d)** *HSD3B1*, 3β-hydroxysteroid dehydrogenase type 1, **(e,f)** *PTGS2*, prostaglandin-endoperoxide Synthase 2, **(g,h)** *PGES*, Prostaglandin E synthase, **(i,j)** *PTGER2*, prostaglandin E receptor 2, **(k,l)** *PTGFR*, prostaglandin F receptor, **(m,n)** *LHCGR*, luteinizing hormone/choriogonadotropin receptor, **(o,p)** *PRLR*, prolactin receptor, **(q,r)** *FSHR*, follicle stimulating hormone receptor, **(s,t)** *GPX4*, glutathione peroxidase 4, **(u,v)** *SOD1*, superoxide dismutase 1. Small letters indicate significant differences between groups, based on pairwise comparison Wilcoxon rank sum test.

All analyzed genes were detectable (Figures 6, 7, Tables S1, S2) in luteal cells of domestic cats, but *PTGS2* and *PGES* were present only at very low levels in LLC.

During SLC culture significant changes were observed for all genes (Figures 6a,c,e,g,i,k,m,o,q,s,u, 7a,c,e,g,i,k,m,o,q,s,u) although the pattern was gene specific. Both steroidogenic enzymes (*CYP11A1* and *HSD3B1*) expressed a similar pattern, with a very strong expression directly after isolation and a significant drop of their RNA abundance after culture (Figures 6a,c, 7a,c). In contrast, enzymes involved in prostaglandin synthesis were characterized by two opposite patterns. Expression of *PTGS2* decreased steadily over the time (Figure 6e), whereas PGES expression increased (Figures 6g, 7g). Hormone receptors (Figures 6i,k,m,o,q, 7i,k,m,o,q) showed comparable patterns to the one described for steroidogenic enzymes. The strongest decrease in expression (274-fold) was observed in *LHCGR* (Figure 6m). The anti-oxidative enzyme *GPX4* increased during the time of cell culture (Figures 6s, 7s) whereas *SOD1* expression strongly decreased (Figures 6u, 7u).

For LLC, no significant changes were measured for genes involved in steroid and prostaglandin synthesis (Figures 6b,d,f,h, 7b,d,f,h). However, a significant increase of gene expression during the culture period was measured for hormone receptors (Figures 6l,n, 7j,n,p,r), except *PTGER2, PRLR, FSHR* in experiment A (Figures 6j,p,r) and *PTGFR* in experiment B (Figure 7l). The two analyzed anti-oxidative enzymes *GPX4* and *SOD1* also increased significantly over time (Figures 6t,v, 7t,v).

## Discussion

We describe for the first time separate cultures of small and large luteal cells in domestic cat, accompanied by hormone and gene expression analysis. All culture systems were performed without any stimulation by potential luteotrophic or luteolytic factors, and therefore present the basis for functional studies on cellular activity of SLC and LLC in domestic cats and for comparative studies in other feline species in the future.

We have performed two series of experiments over different time periods, because there is a lack of information about steroidogenic luteal cells from domestic cat at a very first day in cell culture. Available literature for other species characterize cell cultures at different days (7, 9, 10, 14) including directly after isolation (48).

### Isolation of Small and Large Feline Luteal Cells and Their Characterization

#### Isolation of Steroidogenic Cells

Enzymatic digestion of luteal tissue with collagenase and DNase is the common method of luteal cell isolation (4, 49–52). However, this method generally isolates a mixture of small and large luteal cells, which is then purified by centrifugation through a percoll density gradient (4, 51, 52) or by cell sorting (53). In comparison to bovine CL, feline CL are quite small and cell numbers are very low, so the application of flow cytometry is inappropriate. Fortunately, our modified method based on enzymatic digestion allows us to isolate SLC only.

LLC were exclusively obtained through mechanical method. Isolating luteal cells without enzymatic digestion has rarely been used before. Gregoraszczuk (7) reported the use of mechanical disruption of porcine luteal through a metal strainer, and Srivastava et al. (54) obtained luteal cells without enzymatic digestion based on migration of cells from tissue pieces (7, 54). Using tissue explants, however, requires time to achieve cell migration, creating colonies and monolayer formation and the composition of cell culture might be uncertain. In comparison, our isolation method allows for the isolation of LLC in a very short time and provides a very good way to distinguish them from SLC. To obtain SLC we have modified the method of Arikan and Yigit (14), who had previously described isolation of feline luteal cells. The selectiveness of the enzymatic method is based on the high sensitivity of feline LLC toward enzymatic digestion in contrast to SLC. The extreme fragility of LLC was discussed earlier for rat (11, 55, 56) and was related to the impact of mechanical forces and enzymes on peculiar morphology of LLC with their extremely high number of intracellular lipid droplets and extensive interdigitation (11, 55).

#### Characterization of Freshly Isolated SLC and LLC

The two different isolation protocols described here were the basis for our gene expression analysis in both SLC and LLC, although it must be indicated that the data on relative abundance cannot be compared directly between cell types. Due to the comparably low amount of extracted RNA obtained from LLC, only one reference gene could be used for normalization. In addition, the high variation of measurements between the cell isolates did not allow a more detailed statistical analysis.

Nevertheless, some important conclusions can be inferred from the observed gene expression profiles in the two freshly isolated luteal cell populations of domestic cats. Firstly, all tested genes were found to be expressed, but comparison between the different genes revealed some putative quantitative differences. SLC had a very high level of prostaglandin E2 receptor (*PTGER2*) expression (Figures 6i, 7i), followed by genes of steroidogenic enzymes and the antioxidant system (Figures 6a,c,s,u, 7a,c,s,u). Prostaglandins and their receptors were previously reported to play an important role in the maintenance of carnivore CL (2, 13, 57, 58). Interestingly, both prostaglandin synthases (*PTGS2* and *PGES*, Figures 6e,g, 7e,g) and the receptor for PGF2alpha (*PTGFR*, Figures 6k, 7k) were expressed at considerably lower levels compared to *PTGER2* in SLC. This hints to a more pronounced role of PGE reception in SLC. In LLC, both synthases were almost undetectable (*PTGS2* and *PGES;* Figures 6f,h, 7f,h), while *PTGFR* (Figures 6l, 7l) was at the same expression level as the receptor of *PGE2* (Figures 6j, 7j). This may indicate to a comparable sensitivity of LLC toward both prostaglandins. Previous determination of gene expression in luteal tissue of domestic cats, revealed a decreasing prostaglandin synthesis, once the CL develop from formation toward development/maintenance, accompanied by an increase of prostaglandin receptors, *PTGER4* and *PTGFR* (2).

Also for other analyzed genes, differences in expression patterns between the two steroidogenic luteal cell populations could not previously be identified before using CL tissues. Isolated SLC showed comparatively high expression level for *FSHR* (10–20 times higher, Figures 6q, 7q) in contrast to both, the LH receptor (Figures 6m, 7m) and the prolactin receptor (Figures 6o, 7o). In isolated LLC, the FSH receptor (Figures 6r, 7r) expression level was not different from that of LH (Figures 6n, 7n) and prolactin receptors (Figures 6p, 7p). In our previous studies (unpublished data) on feline luteal tissue, we have determined increasing *FSHR* expression during luteal life span and increasing prolactin receptor expression toward early regression. In contrast, *LHCGR* level decreased toward regression. In other species, luteal LH receptor expression was transiently down regulated in response to the LH-surge, but reactivated in the mid-luteal phase and decreased again with CL regression (59). Domestic cats are induced ovulators and in the case of non-pregnancies a real LH-surge might not occur.

Our two luteal cell populations and their cultures allow for follow-up studies on the response to gonadotrophic stimulation, and provide a better understanding on the role of suggested luteotrophic factors, like LH or prolactin, for the survival of CL; and last but not least, for the physiological persistency of CL described for lynx species (33).

### Functional Behavior of Steroidogenic Luteal Cells During Culture

Although being isolated from similar domestic cats, both established luteal cell culture systems were different with regard to medium composition and cell number inoculated into wells. Our aim was to define marginal suitable culture conditions, which would later allow the study of luteotropic factors. Therefore, outcomes of culture cannot be directly compared and will be discussed separately.

#### Functional Behavior of SLC in Culture

In our cell culture system, SLC morphology was similar to rat luteal cells (11). After seeding, they attached to the cell culture plate within 1 day. Proliferation or cells spreading of feline SLC was ongoing until a confluent monolayer formed at around day 3–4 (Figures 3a,c,e, 4a,c,e). A hint for proliferation is the increasing relative abundance for reference genes per well (data not shown). SLC of domestic cat release less progesterone than LLC (Table 2) per inoculated cells. This activity difference was previously described in sheep (21), pigs (19), cows (8, 20), humans (4), and rats (11). In addition, our feline SLC progressively lost their steroidogenic capabilities over the period of cell culture, as shown by decreasing enzyme activity of 3β-hydroxysteroid dehydrogenase (Figures 5a,c,e) accompanied by a decrease in mRNA expression of steroidogenic genes during culture (Figures 6a,c, 7a,c). In bovines, SLC divided rapidly in the absence of LH, showed signs of mitosis in culture and lost steroidogenic function (60). Arikan et al. (14) described a steady decline of progesterone production between day 3, 5, and 7 of feline luteal cell in culture (14). In contrast, we did not observe a further decrease of P4 production between day 4 and 5 in SLC (Table 2).

Simultaneous determination of steroid production and gene expression as described, might allow us to assess the functionality of the cells in culture. It appears that SLC suffered massively from the isolation procedure and needed at least 1 day to recover within culture. The immediate change in steroid producing activity after inoculation to the culture disk, however, was expected and has been described for rat and bovine luteal cells (11, 61). Unraveling the course of gene activity and hormone production during the first 72 h as described for experiment A, reflected the conversion of SLC *in vivo* (activity at day 0) to probably proliferating (but maybe not non-differentiated) luteal cells between day 1 and 3. Interestingly, we observed a drop in gene expression in some but not all genes. The *PGES* and *GPX4* expression showed a different pattern with a higher expression in cultured cells (Figures 6g,s, 7g,s). In luteal tissue, the *PGES* expression was highest in the formation stage and decreased over luteal life span (2).

Glutathione peroxidase 4, the enzyme which is encoded by the *GPX4* gene, protects cells against membrane lipid peroxidation (62). The expression of this gene did not fall after isolation and in fact increased during the course of culture in SLC. This may have been a reaction to the elevated oxidative stress in the cell culture.

There is evidence that bovine SLC have an ability to differentiate to LLC *in vivo* (16), and this was also described for CL of pseudo-pregnant domestic cats (50). This phenomenon, however, has not previously been described *in vitro*. In the experiments of Hoyer et al. (9), cultured ovine luteal cells significantly increased in size, but this was not described as a cell differentiation. Microscopic analysis of our SLC cultures may indicate that some SLC differentiated into LLC. Around the fourth day of culture, some LLC-like cells were observed occasionally and their number appeared to increase during the culture period (Figures 4c,e). At the beginning of differentiation, cells increased in size, acquired more compact and round shape, accumulated lipid droplets within the cytoplasm, and started to detach from the cell culture dish (Figure 5e). This culminated in a cell type expressing morphology and size characteristics of LLC (Figure 2b). Alternatively those cells can represent cells with diameter >20 μm obtained after isolation, which at the beginning of cell culture flattened on the bottom of a well, but then grew to size typical for LLC and detached from bottom of the well. Another explanation for this may be that cells detached on their way to dying and its bigger size is caused by long time in cell culture, but such a big increase in diameter in dying cells seems unlikely for us.

Enzyme activity for 3ß-hydroxysteroid dehydrogenase of differentiating cells was much more conspicuous (Figure 5e) compared to the SLC, but blocking enzyme activity with trilostane was not as pronounced as that of isolated LLC indicating a difference between these two cell types. Trilostane is a synthetic steroid which selectively inhibits 3ß-hydroxysteroid dehydrogenase (37). Thus, the remaining staining indicates a reduction of formazan by other pathways like the activities of lactate dehydrogenase (63) or of alkaline phosphatase (64, 65). Activity for alkaline phosphatase was previously used to discriminate between theca-derived luteal cells and luteal cells of granulosa cell origin in the pig (66) and the rat (67). In this study we did not specifically select the differentiated luteal cell for expression analysis, but future studies might consider producing and harvesting differentiated SLC to compare their cellular activity with LLC.

#### Functional Behavior of LLC in Culture

The behavior of feline LLC in culture was different from SLC. LLC did not attach to the culture well and did not proliferate. Similar observations regarding lack of proliferation of LLC were previously made in rats (11). Nelson et al. describes also, that LLC of rat do not flatten out completely in culture. It was explained, most probably as the abundance of lipid droplets in LLC. Based on this information and our observation, we suggest that maybe LLC in domestic cat do not attach to the well surface because of high amount of lipid droplets. LLC numbers declined over the period of study, which was reflected by a massive drop in reference gene expression (data not shown). Although it is not clear whether progesterone is released from cellular depots or produced *de novo* (Table 2), intense staining for activity of 3β-hydroxysteroid dehydrogenase after isolation (Figure 5g) and stable gene expression of steroidogenic enzymes support luteal cell activity in surviving cells during culture (Figures 3b,d,f, 4b,d,f). We also observed a steady increase in the expression of the receptors for gonadotrophins, prolactin and PGF2alpha (Figures 6l,n, 7n,p,r). Further experiments are needed to test whether and how LLC will react to LH or FSH stimulation or PGF2alpha supplementation. It has been previously suggested that PGF2alpha might change its function from a luteotrophic agent at the start of pregnancy or luteal cycle toward a luteolytic component around parturition. This was supported by the PGFM profile determined throughout pregnancy in domestic cats (68, 69) and the inability to interrupt pregnancies with prostaglandins before day 33 of pregnancy and the need for extremely high doses thereafter (70, 71). Shille and Stabenfeldt (72) showed that high doses and repeated treatments with PGF2alpha in the first trimester had almost negligible effects (72). During the same time period, the highest level of PGF synthase (*PTGF*) was detected in the feline placenta (73). This, together with our observation that LLC isolated from CL in the stage of development/maintenance express PGF2alpha receptor, points to a luteotrophic function of prostaglandin at the beginning of feline luteal cycle.

## Data Availability Statement

The datasets generated for this study can be found in the GenBank: MH882487, MH882488, MH882490, MH882486, and MH882489.

## Ethics Statement

Our samples are from routine ovariectomies performed in animal clinics and were not related to the purpose of the experiment.

## Author Contributions

MH carried out the study, analyzed and interpreted the data, and compiled the manuscript. BB designed the study with respect to gene expression analysis, partly analyzed and interpreted data, discussed the results, critically revised the manuscript, and finally approved the version for submission. LB performed statistics, proofread for English, critically revised the manuscript, and finally approved the version for submission. KJ designed the study with respect to cell culture, discussed the results, critically revised the manuscript, and finally approved the version for submission. All authors approved the final submitted manuscript.

### Conflict of Interest

The authors declare that the research was conducted in the absence of any commercial or financial relationships that could be construed as a potential conflict of interest.

## Supplementary Material

Figure S1 SLC stained for activity of 3β-hydroxysteroid dehydrogenase SLC at day 2 of cell culture **(a)**; control staining for SLC at day 2 of cell culture **(b)**. In control samples, activity of 3β-hydroxysteroid dehydrogenase was blocked by trilostane.

Table S1 Relative mRNA abundance of genes in small luteal cell cultures (days 1–3 and 3–5) compared to freshly isolated cells (day 0). Results presented as mean values ± standard deviation. *P*-values indicate the result of Kruskal-Wallis test for normalized data, Chi squared—refers to test statistics and df indicates degrees of freedom. Small letters indicate significant differences between groups, based on pairwise comparison Wilcoxon rank sum test. ^*^Indicates that significant differences between groups have the same pattern with expression on day 0 being different from all others days.

Table S2 Relative mRNA abundance of genes in large luteal cell cultures (days 1–3 and 3–5) compared to freshly isolated cells (day 0). Results presented as mean values ± standard deviation. *P*-values indicate the result of Kruskal-Wallis test for normalized data, Chi squared—refers to test statistics and df indicates degrees of freedom. Small letters indicate significant differences between groups, based on pairwise comparison Wilcoxon rank sum test. ^*^Indicates that significant differences between groups have the same pattern with expression on day 0 being different from all others days.
