# Supplementary figures and images for: Functional and Morphological Characterization of Small and Large Steroidogenic Luteal Cells From Domestic Cats Before and During Culture
Source: Front Endocrinol (Lausanne). 2019 Nov 14;10:724. doi: 10.3389/fendo.2019.00724 (PMC6867973; doi:10.3389/fendo.2019.00724)

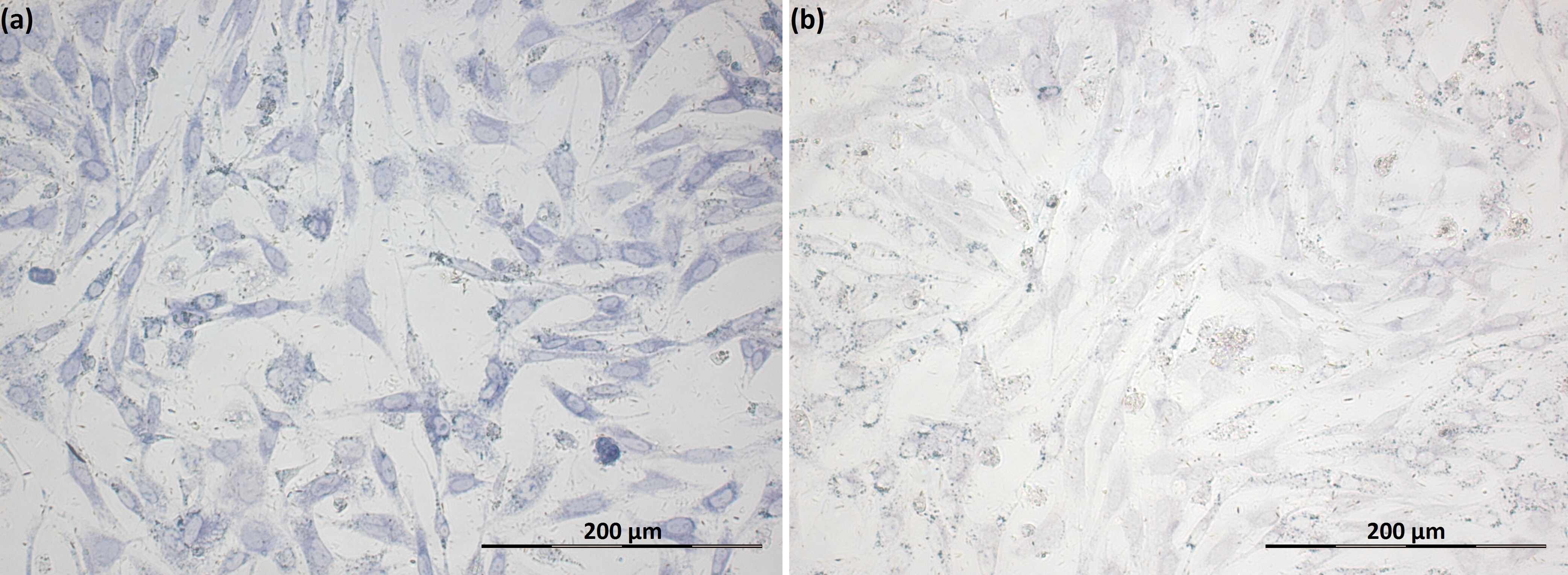

Supplement: Figure S1 — SLC stained for activity of 3β-hydroxysteroid dehydrogenase SLC at day 2 of cell culture (a); control staining for SLC at day 2 of cell culture (b). In control samples, activity of 3β-hydroxysteroid dehydrogenase was blocked by trilostane. [file Image_1.TIF]
