# Supplementary material for: Functional and Morphological Characterization of Small and Large Steroidogenic Luteal Cells From Domestic Cats Before and During Culture
Source: Front Endocrinol (Lausanne). 2019 Nov 14;10:724. doi: 10.3389/fendo.2019.00724 (PMC6867973; doi:10.3389/fendo.2019.00724)
Supplement: Table S2 — Relative mRNA abundance of genes in large luteal cell cultures (days 1–3 and 3–5) compared to freshly isolated cells (day 0). Results presented as mean values ± standard deviation. P-values indicate the result of Kruskal-Wallis test for normalized data, Chi squared—refers to test statistics and df indicates degrees of freedom. Small letters indicate significant differences between groups, based on pairwise comparison Wilcoxon rank sum test. *Indicates that significant differences between groups have the same pattern with expression on day 0 being different from all others days. [file Table_2.DOCX]

**Table S2.** Relative mRNA abundance of genes in large luteal cell cultures (days 1-3 and 3-5) compared to freshly isolated cells (day 0). Results presented as mean values ± standard deviation. p-values indicate the result of Kruskal-Wallis test for normalised data, Chi squared – refers to test statistics and df indicates degrees of freedom. Small letters indicate significant differences between groups, based on pairwise comparison Wilcoxon rank sum test. ^x^ - Indicates that significant differences between groups have the same pattern with expression on day 0 being different from all others days.

|  | **Large Luteal Cells** | | | | | | | |
| --- | --- | --- | --- | --- | --- | --- | --- | --- |
|  | **Gene** | **After isolation** | **Day 1** | **Day 2** | **Day 3** | **P-value** | **Chi-squared** | **df** |
| **Experiment A** | *CYP11A1* | 1995 ± 602 | 2014 ± 865 | 2660 ± 977 | 2527 ± 934 | NS | 3.21 | 3 |
|  | *HSD3B1* | 7682 ± 8690 | 12545 ± 14843 | 12096 ± 14328 | 19579 ± 25689 | NS | 5.61 | 3 |
|  | *PTGS2* | 11 ± 8 | 96 ± 156 | 30 ± 30 | 42 ±38 | NS | 7.25 | 3 |
|  | *PGES/PTGES* | 51 ± 22 | 96 ± 59 | 53 ± 31 | 96 ± 90 | NS | 7.42 | 3 |
|  | *PTGER2* | 345 ± 131 | 220 ± 497 | 128 ± 383 | 765 ± 2296 | NS | 3.14 | 3 |
|  | *PTGFR* | 511 ± 155^x^ | 910 ± 498 | 868 ± 285 | 1045 ± 260 | <0.001 | 19.82 | 3 |
|  | *LHCGR* | 166 ± 102^x^ | 391 ± 328 | 418 ± 292 | 369 ± 249 | <0.05 | 11.76 | 3 |
|  | *PRLR* | 109 ± 32 | 778 ± 1265 | 530 ± 486 | 504 ± 404 | NS | 7.25 | 3 |
|  | *FSHR* | 395 ± 276 | 492 ± 467 | 426 ± 196 | 666 ± 436 | NS | 1.96 | 3 |
|  | *GPX4* | 803 ± 170^x^ | 1335 ± 705 | 1382 ± 527 | 1520 ± 1040 | <0.05 | 11.17 | 3 |
|  | *SOD1* | 5364 ± 1638^x^ | 7624 ± 3174 | 8690 ± 1690 | 9909 ± 4579 | <0.05 | 15.03 | 3 |
|  | **Gene** | **After isolation** | **Day 3** | **Day 4** | **Day 5** | **P-value** | **Chi-squared** | **df** |
| **Experiment B** | *CYP11A1* | 2414 ± 923 | 2772 ± 1199 | 3462 ± 1139 | 2855 ± 1535 | NS | 5.22 | 3 |
|  | *HSD3B1* | 4991 ± 7056 | 7118 ± 10688 | 6314 ± 8713 | 6846 ± 11245 | NS | 0.81 | 3 |
|  | *PTGS2* | 8 ± 4 | 10 ± 15 | 51 ± 84 | 85 ± 138 | NS | 3.91 | 3 |
|  | *PGES/PTGES* | 59 ± 40 | 82 ± 86 | 115 ± 84 | 124 ± 91 | NS | 4.92 | 3 |
|  | *PTGER2* | 358 ± 249^x^ | 308 ± 924 | 0 ± 0 | 122 ± 367 | <0.002 | 15.40 | 3 |
|  | *PTGFR* | 735 ± 82 | 715 ± 281 | 1348 ± 1330 | 992 ± 412 | NS | 6.16 | 3 |
|  | *LHCGR* | 137 ± 76^x^ | 433 ± 173 | 684 ± 458 | 680 ± 350 | <0.001 | 19.99 | 3 |
|  | *PRLR* | 98 ± 38^x^ | 234 ± 190 | 466 ± 412 | 413 ± 244 | <0.001 | 17.56 | 3 |
|  | *FSHR* | 396 ± 193^a^ | 1006 ± 910^a^ | 1374 ± 898^b^ | 2064 ± 2223^b^ | <0.05 | 13.75 | 3 |
|  | *GPX4* | 834 ± 154^a^ | 1607 ± 1050^a^ | 2297 ± 914^b^ | 1346 ± 569^a^ | <0.05 | 15.76 | 3 |
|  | *SOD1* | 12239 ± 6784^a^ | 17007 ± 9848^a^ | 23382 ± 10966^b^ | 19820 ± 11765^a^ | <0.05 | 12.3 | 3 |
